# A Phenotypic Point of View of the Adaptive Radiation of Crested Newts (*Triturus cristatus* Superspecies, Caudata, Amphibia)

**DOI:** 10.1155/2012/740605

**Published:** 2012-01-16

**Authors:** Ana Ivanović, Georg Džukić, Miloš Kalezić

**Affiliations:** ^1^Institute of Zoology, Faculty of Biology, University of Belgrade, 11000 Belgrade, Serbia; ^2^Department of Evolutionary Biology, Institute for Biological Research “Siniša Stanković”, University of Belgrade, 11000 Belgrade, Serbia

## Abstract

The divergence in phenotype and habitat preference within the crested newt *Triturus cristatus* superspecies, examined across different ontogenetic stages, provides an excellent setting to explore the pattern of adaptive radiation. The crested newts form a well-supported monophyletic clade for which at least the full mitochondrial DNA phylogeny is resolved. Here we summarise studies that explored the variation in morphological (larval and adult body form, limb skeleton, and skull shape) and other phenotypic traits (early life history, developmental sequences, larval growth rate, and sexual dimorphism) to infer the magnitude and direction of evolutionary changes in crested newts. The phenotypic traits show a high level of concordance in the pattern of variation; there is a cline-like variation, from *T. dobrogicus*, via *T. cristatus*, *T. carnifex*, and *T. macedonicus* to the *T. karelinii* group. This pattern matches the cline of ecological preferences; *T. dobrogicus* is relatively aquatic, followed by *T. cristatus*. *T. macedonicus*, *T. carnifex*, and the *T. karelinii* group are relatively terrestrial. The observed pattern indicates that phenotypic diversification in crested newts emerged due to an evolutionary switch in ecological preferences. Furthermore, the pattern indicates that heterochronic changes, or changes in the timing and rate of development, underlie the observed phenotypic evolutionary diversification.

## 1. Introduction

Exploring patterns of phenotypic variation during ontogeny and phylogeny is fundamental to gaining insights into the processes of evolutionary diversification, including the mechanisms of speciation. The connection between development, evolutionary history, ecology, and morphology has intrigued evolutionary biologists for over the 150 years since Darwin first published his ideas about natural selection [1]. This is largely due to the idea that phenotypic evolution might be explained by changing or truncating the ancestral ontogeny, for which the characteristics can be inferred through phylogenetic analyses [2–4].

Within a monophyletic group of closely related species, it is expected that shared evolutionary history is reflected by phenotypic similarity, due to a shared developmental basis inherited from a common ancestor. Adaptive radiation and morphological divergence are usually attributed to differential selection acting upon geographical populations. In other words, “ecological opportunity” could lead to adaptive radiation [5, 6]. When phenotypic divergence is paralleled in multiple complex phenotypic traits with separate developmental pathways, this is indicative of adaptation to differential environmental selection pressures [6–9]. This line of reasoning is especially strong in situations where phenotypic variation correlates with different ecological demands.

The crested newts (*Triturus cristatus* superspecies) are an example of an adaptive radiation. Crested newts have been used as model organisms in various studies in evolutionary biology, including the processes and outcomes of speciation [10–14]. The phenotypic and ecological divergence in crested newts, examined across different ontogenetic stages, provides an excellent model to explore the tradeoff between shared evolutionary history and divergent functional requirements within an adaptive radiation.

Here we summarise data from previous studies on crested newts. The resulting framework can be used to evaluate how developmental and functional processes have impacted phenotypic evolution. 

## 2. About Crested Newts

Crested newts belong to the group of the Modern Eurasian newts [33]. Crested newts have a biphasic life cycle with aquatic larvae that metamorphose into terrestrial juveniles and as adults return to the water annually to breed [34, 35]. Crested newts have low mobility, a small dispersal range, and strong philopatric behaviour, which promote genetic isolation. The crested newts form a well-supported monophyletic clade of closely related species for which the molecular phylogeny has been largely resolved [11, 14, 33, 36]. According to current taxonomy, this group consists of six species: *T. cristatus*, *T. dobrogicus*, the closely related species *T. carnifex* and *T. macedonicus* [11], and two species that belong to the so-called *T. karelinii* group, *T. karelinii* and *T. arntzeni*. Mitochondrial DNA studies showing three distinct clades within the *T. karelinii* group [14, 15] illustrate that the taxonomy of the *T. karelinii* clade is as yet unsettled. In an attempt to simplify, we will refer to these newts as the *T. karelinii *group.

**Figure 1:**
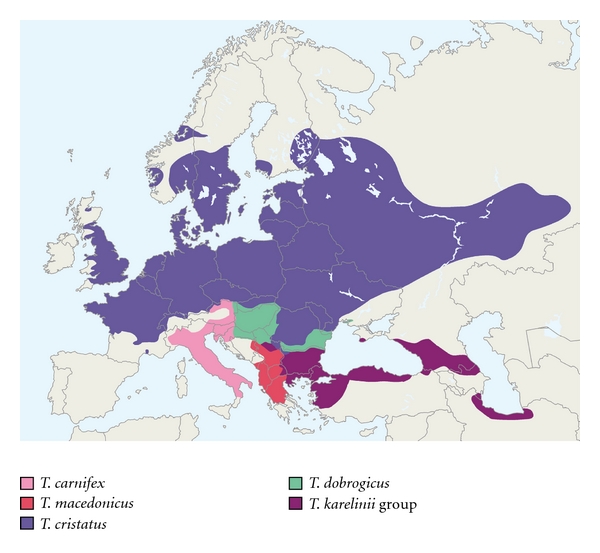
The approximate ranges of the crested newt species. The distribution of *Triturus carnifex*, *T. macedonicus*, *T. cristatus*,* T. dobrogicus,* and the *T. karelinii* group, illustrated after [14–17].

The range of the crested newts spans most of Europe and adjacent Asia (Figure 1). The nominotypical species (*T. cristatus*) is the most widely distributed over much of Europe. *T. dobrogicus* is confined to the Pannonian area and the Danube Delta and Dobrugea Plain. The other species have a more southern distribution, restricted to the Apennine Peninsula and the northeast Balkan Peninsula (*T. carnifex*), the western Balkan Peninsula (*T. macedonicus*) and the eastern Balkan Peninsula, Asia Minor, Crimea, Caucasus, and southern shore of the Caspian Sea (*T. karelinii *group). The species have a parapatric distribution and a potential to interbreed along the contact zones, especially in the Balkan region [37, 38].

**Figure 2:**
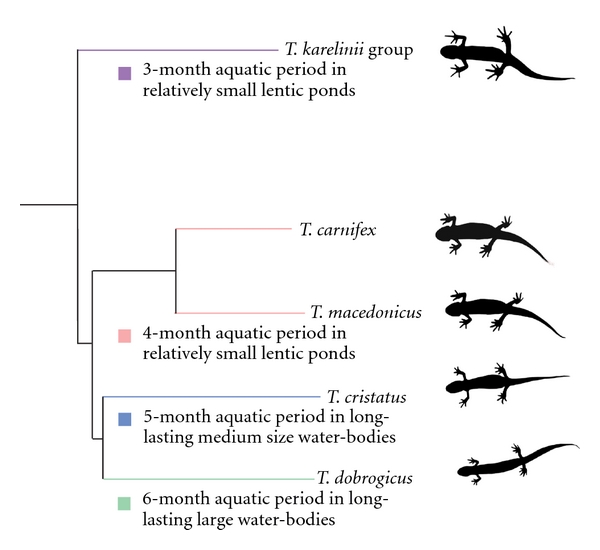
The phylogeny, the habitat preferences and the ecological demands of the crested newt species [18, 19]. To infer the direction of evolutionary changes among crested newt species the latest, most complete phylogenetic analysis [14] was used. The branch lengths are proportional to the number of base substitutions per site [14].

The crested newt species differ in their ecological demands [18, 19]. The most aquatic is *T. dobrogicus*, followed by *T. cristatus. *Generally,* T*. *macedonicus*, *T. carnifex* and the *T. karelinii* group are the more terrestrial species. *T. dobrogicus* inhabits permanent and/or long-lasting, large, stagnant water bodies. *T. cristatus* occupies mostly long-lasting medium-sized water bodies. The other crested newt species are associated with relatively small lentic ponds with variable hydroperiods [18, 37]. The duration of the aquatic phase is directly related to their habitat and varies from a short, three months in the *T. karelinii *group up to six months in *T. dobrogicus* (Figure 2). It is worth to note that the evolution of habitat preferences of crested newts still needs to be addressed properly.

The most notable characteristic of the crested newts' origin is that their evolutionary splitting occurred within a short-time span, which indicates that there was a burst of speciation rather than a prolonged process of speciation [11, 14, 36]. This is typical for adaptive radiations. Ecologically based, spatially heterogeneous selection, coupled with limited migration, can result in rapid phenotypic diversification [39, 40]. Such a scenario presumes the existence of divergent ecological conditions, as well as a low magnitude of phenotypic and genetic correlations. A high level of phenotypic divergence could be achieved even under substantial hybridisation and gene flow [41].

## 3. Interspecific Variation in Phenotypic Traits

### 3.1. Adult Body Form

The term “body form” refers to the robust morphological features of an organism's external morphology and encompasses both size- and shape-related characteristics. Body form can differ between species, but also between groups of species. In tailed amphibians, adaptation to an aquatic life is usually related to body elongation and limb reduction, which may increase swimming performance. A compact body and robust limbs are linked to a terrestrial life and a lack of passive buoyancy.

Crested newts show a range body forms from a slender body and short limbs in *T. dobrogicus, *via *T. cristatus, *and* T*. *macedonicus* and *T. carnifex* to a short body and long limbs in the *T. karelinii* group [37, 42]. The ancestral phenotype, a large body with a short trunk and a wide head, characterises the *T. karelinii *group. The species *T. carnifex* and *T. macedonicus* have a large body and wide head accompanied by mild body elongation. The most derived phenotype includes body size reduction and more pronounced body elongation in *T. cristatus *and, especially, in *T. dobrogicus* (Figure 2). Body elongation in these newts is reflected in the modal number of rib-bearing vertebrae: 13 in *T. karelinii* group, 14 in *T. macedonicus* and *T. carnifex*, 15 for *T. cristatus,* and 16 or 17 in the most elongated *T. dobrogicus* [14, 37, 43, 44].

### 3.2. Ontogeny of Body Shape

**Figure 3:**
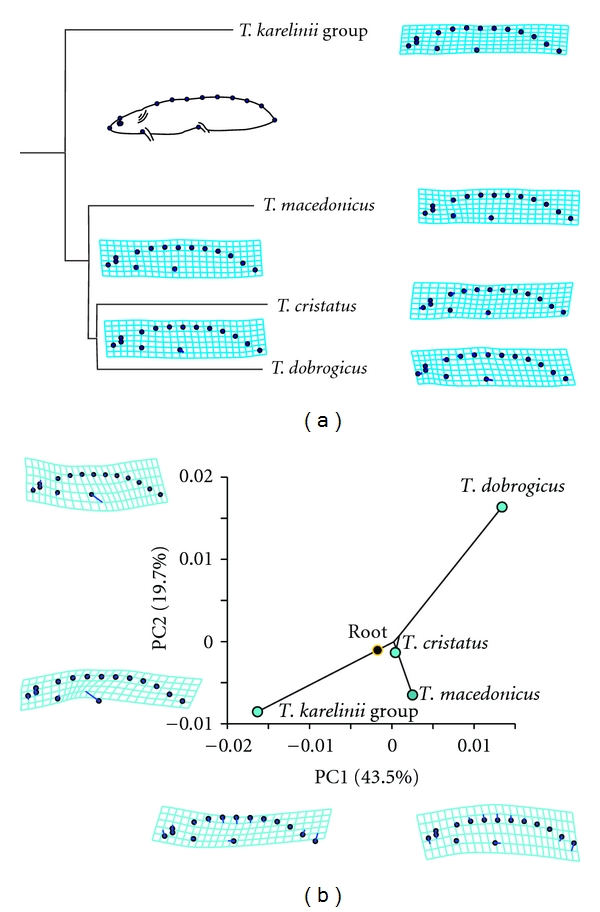
The shape changes of 90-day-old larvae mapped on the crested newt phylogeny (a) and the phylogeny superimposed in the morphospace defined by the first two principal axes (b). To capture larval shape, both landmarks and semilandmarks presenting the shape of dorsal caudal fin at larvae and tail shape were used [20]. We applied a procedure for mapping the geometric morphometric data onto a known phylogeny [21–23]. The criteria of squared-change parsimony (weighted by divergence time or molecular change on the respective branches of the tree) were used for reconstructing the values of the internal nodes of the phylogeny from the shape averages of the terminal taxa [24–27]. We used the generalised method of least squares [26, 28] to find values for the internal nodes. The sum of squared changes along the branches is minimised over the entire phylogeny. We applied evolutionary principal component analysis [21], and the ordination of mean shapes in the space of the first two principal axes is presented. The thin-plate spline deformation grids that illustrate lateral larval shape changes correlated with the first and the second axis are presented [22]. The analyses and the visualisation of shape changes in the evolutionary morphospace were performed using the MorphoJ software [29].

The ontogenetic niche shift and transition between the aquatic and terrestrial habitats is coupled with metamorphosis and an overall change in the relationship between the individual and its environment. Therefore, two different sets of adaptations and constraints during growth could shape the ontogenetic trajectories of crested newts and affect their phenotypic diversification. The analysis of ontogenetic shape changes and changes in developmental rate [20] gives insight into the processes of the evolutionary diversification of the crested newts. The four analysed species of crested newts (*T. dobrogicus, T. cristatus, T. macedonicus,* and* T. arntzeni *(*T. karelinii* group), differ in size and shape as larvae, at least when the larval body form is fully developed (i.e., midlarval stage). The ontogenetic trajectories of larval shape diverge in both the direction and the rate of shape changes along species-specific trajectories [20]. The species significantly differ in their developmental rate of larval shape, except for *T. cristatus *and* T. dobrogicus*, which are similar*. T. dobrogicus* clearly differed from the other species in having a higher and wider caudal fin, while *T. arntzeni *(*T. karelinii *group) has the most elongated larvae with the lowest tail fin (Figure 3). Based on the assumption that the shape of caudal fins is of high adaptive significance for larvae, it is tempting to hypothesise that the two larval shape types represent aquatic ecological adaptations of the two species groups of crested newt.

Contrary to clear discrimination between species in larval body shape, at the juvenile stage just after metamorphosis, the species converge on a similar body shape [20]. After that stage, crested newt species apparently diverge toward the adult body shape. The differences in body shape that we found may indicate that the body forms of larvae and adult individuals are subject to selection in both the aquatic and terrestrial environments, resulting in the same pattern of interspecific differences, despite the possibility of two distinct sets of constraints [45]. 

### 3.3. Limb Size, Ossification Level, and the Pattern of Morphological Integration

**Figure 4:**
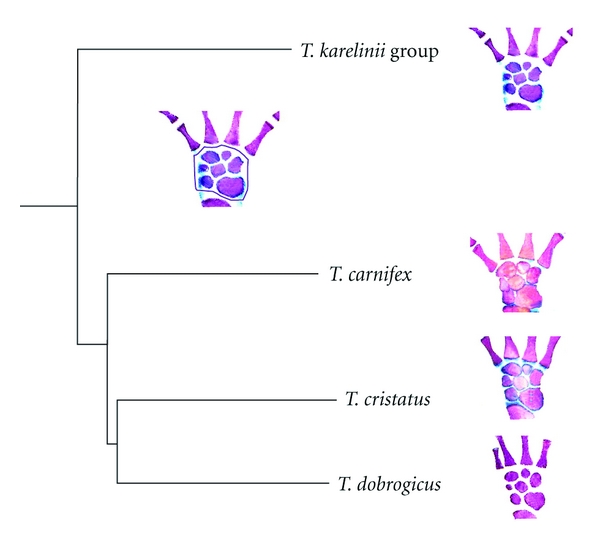
The forelimbs of four crested newt species that illustrate differences in “bone-packaging” [30]. Bones are coloured in red, cartilage in blue, and the surrounding soft tissue is clear and semitransparent. The statistically lower ossification level and concomitant loose “bone packaging” characterise *T. dobrogicus. *

A morphometric analysis of the limb skeleton of four crested newt species (*T. dobrogicus, T. cristatus, T. carnifex, *and* T. arntzeni *(*T. karelinii *group)) showed that although they differ in the size of skeletal elements (stylopodium, zeugopodium, and third metapodial element), they all shared common allometric slopes [30]. A similar relationship between limb skeleton size and body size could indicate a conservative direction of ontogeny [30]. However, a lateral shift in the species-specific allometries of *T. dobrogicus *and *T. cristatus* indicates evolutionary changes in the allometric trajectories. The lateral shift could indicate that heterochronic changes underlie the observed morphological variation [3]. Moreover, the *T. dobrogicus *manus has a significantly lower ossification level and concomitant loose “bone packaging” compared with the other species (Figure 4), which could be a result of the heterochronic changes [46, 47].

**Figure 5:**
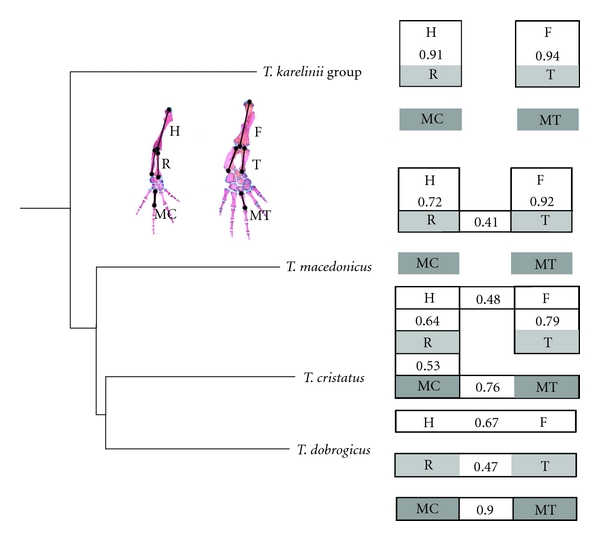
Graphical model of the significant partial correlations illustrated as boxes between limb elements (*P* < 0.05) of adult individuals [13]. H: humerus; R: radius; MC: metacarpal; F: femur; T: tibia; MT: metatarsal. Partial correlations measure a correlation between two variables that are independent of information from the other variables in the correlation matrix. The significance of partial correlations was assessed using an information theoretic measure known as the edge exclusion deviance (EED) and the *χ* ^2^ distribution: EED = −*N*ln⁡⁡(1 − *ρ*_*ij*{*K*}_^2^), where *N* is the sample size, and *ρ* ^2^_*ij*{*K*}_ is the partial correlation coefficient between variables *i* and *j* [31]. The two variables were conditionally independent when the EED value was less than 3.84 (corresponding to *P* = 0.05, df = 1 from the *χ* ^2^ distribution). In *T. dobrogicus*, significant edges were present only between homologous limb elements. This finding is in opposition to *T. arntzeni *(*T. karelinii* group) which had high partial correlations within limbs between the stylopod and zeugopod elements. *T. arntzeni* (*T. karelinii* group) had a stronger correlation of skeletal elements within the limbs than between fore- and hindlimbs. The partial correlations between limb elements in *T. cristatus* and *T. macedonicus* were intermediate with regard to the previous species, with variable significant edges between homologous and within-limb elements.

Limbs, as serially homologous structures, share a strong developmental basis. The shared genetic factors (e.g., *Hox* patterning genes) are intrinsic to the covariation among the homologous structures within limbs (e.g., radius and tibia or humerus and femur) and the overall morphological integration [48, 49]. Epigenetic factors, such as function, also could have an impact on limb integration. In empirical studies, the expression of functional and developmental interdependencies in the patterns of integration could be estimated. If covariation between the homologous parts of the fore- and hindlimbs is stronger than the covariation of skeletal elements within the limbs, then developmental constraints prevail over functional determinants [49–51]. Two opposing correlation patterns were observed [13] in the more terrestrial species, the homologous limb elements were less correlated, and the within-limb elements were more correlated, whereas, in the aquatic species, the reverse pattern occurred (Figure 5). All of these results indicate that function appears to be the covariance-generating factor that has shaped the patterns of morphological integration of crested newt limbs.

### 3.4. Skull Shape and Ontogenetic Skull Shape Changes

**Figure 6:**
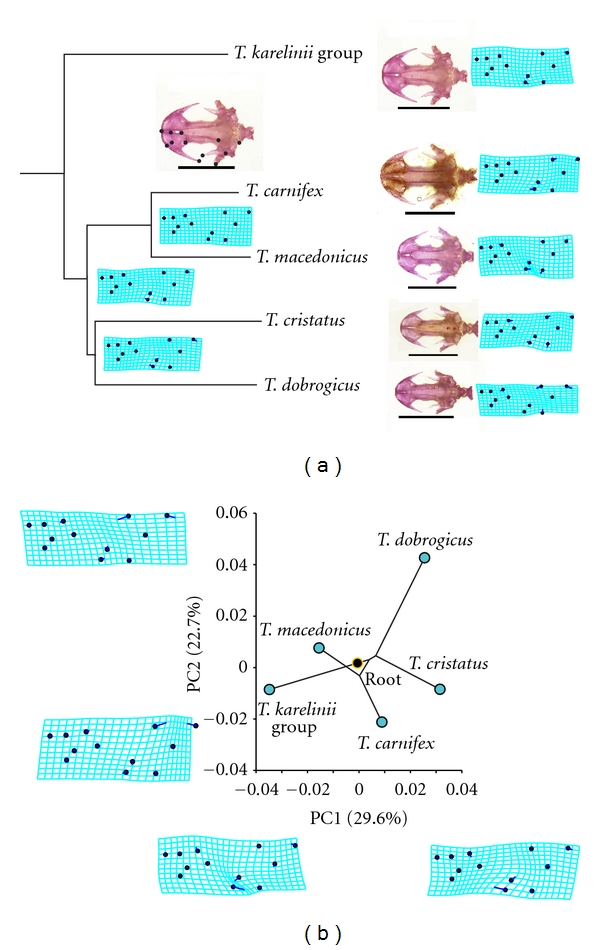
Skull shape changes mapped on the crested newt phylogeny (a), and the phylogeny superimposed in the morphospace defined by the first two principal axes (b). To calculate mean shape, we used a subset consisting of male specimens from population samples used for the study of variation in crested newt skull shape [32]. To visualise the changes of ventral skull shape along the crested newt phylogeny, we applied a procedure for mapping geometric morphometric data onto a known phylogeny [23]. The criteria of squared-change parsimony (weighted by divergence time or molecular change on the respective branches of the tree) were used for reconstructing the values of the internal nodes of the phylogeny from the shape averages of the terminal taxa [24–27]. We used the generalised method of least squares [26, 28] to find values for the internal nodes. The sum of squared changes along the branches is minimised over the entire phylogeny. We applied evolutionary principal component analysis [21], and the ordination of the mean shapes of five *Triturus* species in the space of the first two principal axes is presented. The thin-plate spline deformation grids that illustrate skull shape changes correlated with the first and the second axis are presented [22]. The analyses were performed using MorphJ software [29].

Crested newts differ in skull shape [32]. The visualisation of the phylogeny superimposed in the morphospace, and the positions of the internal nodes in the phylomorphospace (Figure 6) indicate that most of the shape changes occurred along species-specific branches. *T. dobrogicus *markedly diverged in skull shape as reflected by a more slender and elongated skull (Figure 6). The advantages of such morphology might be better locomotion in aquatic habitats due to a more streamline body shape but with the possible disadvantage of reduced abilities of suction feeding. The similarities in skull shape of *T. macedonicus* and *T. arntzeni *(*T. karelinii* group) probably reflect a symplesiomorphy.

**Figure 7:**
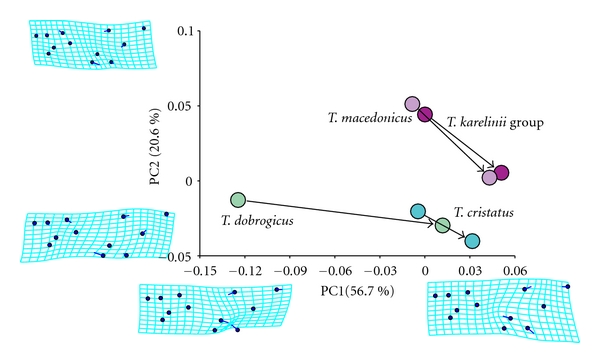
Postmetamorphic skull shape changes. The analysed sample of juveniles at the stage just after metamorphosis was obtained from a laboratory breeding experiment [53]. The sample of juveniles consisted of 10 specimens of *T. dobrogicus*, *T. macedonicus,* and *T. arntzeni *(*T. karelinii* group) and 5 specimens of *T. cristatus*. A general procrustes analysis [55–57] was performed for the entire sample of juvenile and adult specimens of the four analysed species. We calculated the mean shape for each stage and species and performed exploratory, principal component analysis. The positions of species- and stage-specific mean shapes in the morphospace defined by the first two principal axes are given. An arrow connecting the means for juveniles and those for adult specimens in the morphospace defined by the first two principal axes visualises the direction and the amount of shape changes during skull ontogeny.

The analysis of the ontogenetic trajectories of skull shape changes between juveniles just after metamorphosis and adults [12] indicate that *T. dobrogicus *has the highest rate of cranial shape change during postmetamorphic growth, as well as a distinctive ontogenetic allometric trajectory compared with the other three analysed species (*T cristatus, T. carnifex, *and *T. arntzeni *(*T. karelinii* group)). To visualise the direction and amount of shape changes during crested newt skull shape ontogeny (Figure 7), we performed an additional analysis using a larger sample of juveniles than available for the study of ontogenetic shape changes [12]. The slope and the amount of ontogenetic shape change of *T. dobrogicus* clearly diverged from the other species. It is interesting to note that the changes in skull shape that clearly separate *T. dobrogicus* and *T. cristatus *from *T. macedonicus* and the *T. karelinii *group are shared in both analysed stages: the juvenile stage just after metamorphosis and the adult stage (Figure 7).

### 3.5. Early Ontogeny

Comparative study of crested newt development and early life history traits, such as egg characteristics, developmental rate, survival rate, and duration of the embryonic period [52, 53] produced data valuable for understanding the forces shaping adaptation and evolutionary diversification. In vertebrates, vitellus size appears to be one of the key life-history traits reflecting maternal input and could affect the development rate and the size and stage of larvae at hatching [54]. The vitellus size and thickness of the mucoid capsule that protects from injury, fungal infestation, and ultraviolet-B radiation were investigated in four crested newt species (*T. macedonicus*, *T. cristatus*, *T. dobrogicus, *and *T. arntzeni *(*T. karelinii* group)) [52]. Larger crested newt females tend to produce eggs with larger vitelluses. Although the studied species shared a common allometric slope of the egg size versus body size relationship, the species differed in the egg size, which appeared to be a species-specific life-history trait with a cline-like distribution; *T. dobrogicus *has the smallest eggs, the members of the *T. karelinii *group and *T. macedonicus *have the largest eggs, and *T. cristatus *has intermediate-sized eggs.

The crested newts are similar with respect to basic developmental traits (no differences in developmental sequences and survival rates). However, there is a significant variation in the developmental rate. Generally, the developmental rate highly depends on environmental factors, especially temperature. Under experimental conditions [53], *T. dobrogicus *appears to be the outlier species, particularly in comparison to *T. arntzeni *(*T. karelinii *group) and *T. macedonicus*, which have the longest developmental period [53]. Also, there are differences in the pattern of correlation amongst life-history and developmental traits. The comparisons of phenotypic correlation matrices based on eleven life history and developmental traits [53] revealed that *T. dobrogicus *have a correlation pattern similar to *T. cristatus* and *T. macedonicus. T. arntzeni *(*T. karelinii* group) has a similar correlation pattern to *T. macedonicus*, but there are no similarities in the matrix correlation pattern compared to the other two species. 

## 4. Adaptive Radiation Pattern

Adaptive radiation refers to the evolution of ecological and phenotypic diversity within multiple lineages [41]. There are four criteria to detect an adaptive radiation [40]; common ancestry, rapid radiation, environmental correlation, and trait utility. In crested newts, common ancestry and rapid radiation can be readily inferred from phylogenetic analysis. Also, there is a perfect match between the patterns of interspecific differentiation in phenotypic traits and ecological preferences. However, no clear relationship between evolution of body form and locomotor function in newts was found [10]. The onset of adaptive radiation often requires, in addition to the existence of a new habitat, the possession of a key innovation that allows rapid adaptation in novel ecological settings [58].

The observed pattern of differences in the crested newts' phenotypic characteristics shows that *T. dobrogicus* is in any respect the most derived species. *T. dobrogicus* has (1) the most elongated body and the largest number of rib-bearing vertebrae [14, 42]; (2) a significantly different size, ossification level, and pattern of morphological integration of limbs [13, 30]; (3) a marked difference in skull shape [32], including the direction and rate of ontogenetic shape changes [12]; (4) the smallest vitellus [52]; (5) peculiarities in life history traits [53]; (6) a distinct pattern of sexual dimorphism of morphometric traits (the sexual dimorphism in body size is absent in *T. dobrogicus*, while in other species females are the larger sex [59]). However, from this long list of diverged phenotypic traits that characterise* T. dobrogicus*, a key innovation cannot yet be clearly recognised.

## 5. Possible Mechanism of the Crested Newts' Evolutionary Diversification in Phenotypic Traits

External restrictions imposed by ecology had a strong influence on the crested newts' phenotypic divergences, including development. In our view, the evolutionary diversification of phenotypic traits in crested newts was driven by ecological speciation. (An additional separation between crested newts might occur through parapatric speciation, in which populations diverge with some gene flow [60].)

The pattern of divergence in developmental rate and correlation pattern between several early life-history and developmental traits [52, 53] indicates that crested newt evolution seemed to be accompanied by a significant ecological diversification and by labile development patterning, including differences in developmental timing. Heterochrony, differences in the sequence of developmental events, the timing and the rates of development, are often invoked as causes that underlie observed phenotypic evolutionary changes [61]. Crested newts are prone to heterochronic changes [62]. The data collected so far suggest that heterochronic changes in early ontogeny can lead to the lateral transposition of the *T. dobrogicus* ontogenetic trajectories, as previously suggested for the cranial shape [12] and allometric limb skeleton trajectories [30].

## 6. The Ecological Shift Drives the Evolution of Phenotypic Traits in the Crested Newts

Natural selection related to shifts in ecology (e.g., invasion of new habitats) can lead to extremely rapid divergence [63]. The new, colonising populations are particularly likely to diverge, especially because they are usually small and likely to be genetically altered. Recently, it has been proposed that new species generally emerge from single events (e.g., changes in environments), and that ecological adaptation promotes reproductive isolation and speciation [58, 64, 65]. Also, a growing body of research demonstrates a link between rapid ecological divergence and speciation [66].

We advocate the hypothesis that the phenotypic diversification in crested newts emerged due to an evolutionary switch in ecological preferences [53]. The phenotypic characteristics of crested newts could have evolved over a short-time span during which the main crested newts phylogenetic lineages diverged in the central Balkans [11, 43]. The main ecological shift of *T. dobrogicus* (and less apparently of *T. cristatus*) could happen due to the availability of aquatic habitats on the Central Balkans [67, 68]. The subsequent phenotypic evolution of *T. dobrogicus* could be amplified through ecological selection, when the species occupied extensive lowland floodplains along the present-day Danube River and its tributaries which were covered with swamps and marshes. This event dated back to Pliocene when the Pannonian Sea dried out, more than three million years after *T. dobrogicus* had separated from other crested newts' species [14]. Similar changes might have occurred when *T. cristatus* spread across the European plains after Pleistocene glaciations. If so, adaptive phenotypic radiation in crested newts appears as an extension of the initial process of speciation.

For both *T. dobrogicus* and *T. cristatus, *the acquiring of new habitats by phenotypic diversification was not accompanied by additional lineage differentiation. Most likely, the high connectivity of large lowland water bodies, distributed in areas affected by glaciations, prevented the long-term partitioning of populations and thus inhibited speciation [69, 70]. In contrast, within the *T. karelinii* group, subdivision across a heterogeneous landscape, especially those in glacial refuges, promotes geographical isolation and thus speciation [15, 69].

## 7. Conclusions

The morphologically and ecologically diversified species of crested newts exhibit a cline-like variation pattern in phenotypic traits, with *T. dobrogicus* and the *T. karelinii* group on the opposite poles and *T. cristatus* as an intermediate species, while *T. carnifex* and *T. macedonicus* are close to the *T. karelinii* group. This pattern matches the cline of the species' ecological preferences indicating that phenotypic diversification in crested newts emerged most likely due to an evolutionary switch in ecological preferences. The patterns of variation also indicate that heterochronic changes underlie the observed phenotypic evolutionary changes.
